# Shed Syndecan-4 and Its Possible Roles in Osteoarthritis

**DOI:** 10.3390/biomedicines13051037

**Published:** 2025-04-25

**Authors:** Kangping He, Haozhe Ren, Xiaohua Chen, Feng He, Yueying Zhang, Hongyun Zhang, Feifei Li, Shibin Yu

**Affiliations:** 1 State Key Laboratory of Oral & Maxillofacial Reconstruction and Regeneration, National Clinical Research Center for Oral Diseases, Shaanxi International Joint Research Center for Oral Diseases, School of Stomatology, The Fourth Military Medical University, Xi’an 710032, China; 2 School/Hospital of Stomatology, Lanzhou University, Lanzhou 730000, China; 3 Key Laboratory of Shaanxi Province for Craniofacial Precision Medicine Research, Department of Orthodontics, College of Stomatology, Xi’an Jiaotong University, Xi’an 710049, China

**Keywords:** osteoarthritis, cartilage, heparan sulfate proteoglycan, syndecan-4, shed syndecan-4

## Abstract

The specific pathogenesis of osteoarthritis (OA) remains not fully understood. As a transmembrane heparan sulfate proteoglycan, syndecan-4 (SDC4) has been proven to play an important role in the development of OA. Notably, the extracellular domain of SDC4 can be cleaved by proteolytic enzymes, leading to the release of shed SDC4 (sSDC4), which subsequently regulates various biological processes in an autocrine or paracrine manner. This review analyzed 97 publications (1987–2025) from Pubmed and the Web of Science Core Collection using specific key words (syndecan-4, shed syndecan-4, and osteoarthritis), providing a comprehensive overview of the current research on sSDC4, including its shedding enzymes and specific cleavage sites, as well as the factors and mechanisms that influence SDC4 shedding. Furthermore, it summarizes the functions of both sSDC4 and its remaining membrane-bound domain. Finally, the roles of sSDC4 in OA are discussed to identify potential therapeutic targets and explore new strategies for the treatment of OA.

## 1. Introduction

Osteoarthritis (OA), a chronic degenerative joint disease, is characterized by pain and impaired joint mobility, significantly reducing quality of life and imposing economic burdens on patients and society [1]. It is estimated that over 500 million individuals worldwide suffer from OA [2]. However, there is no effective disease-modifying therapy for this prevalent and debilitating condition. Current management strategies for OA, including pharmacological interventions, physical therapy, and surgical procedures, provide palliative care but do not halt disease progression [3,4]. A definitive cure for OA remains elusive. Therefore, it is essential to understand and identify the key signaling pathways and molecules that play critical roles in the initiation and progression of OA.

The primary pathological changes in OA encompass the progressive degradation of articular cartilage, abnormal remodeling of subchondral bone, osteophyte formation, and synovial inflammation [5]. Considerable effort has been directed towards understanding the molecular mechanisms of OA and developing a fundamental treatment aimed at preventing the condition or repairing damaged articular cartilage. Articular cartilage is predominantly composed of the extracellular matrix (ECM) and chondrocytes. The interaction between the ECM and chondrocytes is crucial for maintaining articular cartilage homeostasis. The ECM, composed of type II collagen (COL II), proteoglycans (PGs), hyaluronic acid (HA), and chondroitin sulfate (CS), regulates chondrocyte differentiation, metabolism, matrix remodeling, and survival through integrin-mediated signaling. In OA, increased ECM degradation, mediated by enzymes like matrix metalloproteinases (MMPs) and aggrecanases, disrupts ECM components. Chondrocytes respond to these changes by releasing inflammatory cytokines, such as IL-1β and TNF-α, which exacerbate inflammation and ECM breakdown, while also increasing chondrocyte death and furthering cartilage degeneration [6]. The degradation of the ECM and the chondrocyte death are central pathological events in OA, making the interaction between the ECM and chondrocytes a critical factor in maintaining articular cartilage homeostasis [6].

Heparan sulfate proteoglycans (HSPGs), which are type I transmembrane proteoglycans that interact with both the ECM and chondrocytes, contain complex highly sulfated carbohydrate chains covalently attached to their core proteins [7]. HSPGs play essential roles in the interaction between the ECM and chondrocytes. Key members of the HSPG family include the multidomain proteoglycans syndecan (SDC) 1–4 [8]. Among them, SDC4 is the smallest, with a molecular weight of approximately 22 kDa [9]. As a ubiquitous cell surface receptor, SDC4 modulates cell proliferation, migration, mechanotransduction, and endocytosis [10]. Previous studies have demonstrated that SDC4 can modulate the secretion of inflammatory cytokines, which is regulated by low-density lipoprotein receptor-related protein (LRP) in metabolic processes [11] and critically participates in mechanotransduction pathways [12]. As a transmembrane proteoglycan, SDC4 primarily exerts localized effects within the articular microenvironment. In OA, upregulated SDC4 expression promotes disease progression through microRNA-mediated regulation by elevating miR-96-5p to suppress hypoxia-inducible factor 2 alpha (HIF-2α) expression, thereby inhibiting chondrocyte hypertrophy and matrix degradation [13], as well as through the activation of the mitogen-activated protein kinase (MAPK) signaling pathways of extracellular regulated protein kinase (ERK) 1/2 and p38 [14]. Notably, intra-articular injection of SDC4 antibody has been observed to ameliorate cartilage degeneration [14]. These findings collectively suggest that SDC4 participates in multiple pathological pathways in OA, establishing it as a pivotal molecular target with significant therapeutic potential. It is precisely this multifaceted involvement in OA pathogenesis that motivated our investigation of SDC4 as a promising therapeutic strategy. However, its detailed mechanism remains unclear. Interestingly, the extracellular domain (ectodomain) of SDC4, which is normally shed by proteolytic enzymes at low levels under physiological conditions, exhibits increased shedding in response to pathological stimuli such as inflammation or mechanical stress [15]. The soluble shed syndecan-4 (sSDC4) ectodomain, released into the ECM, retains the ability to bind growth factors and can function as an agonist or antagonist in an autocrine or paracrine manner [16]. It can also enter the synovial fluid and blood and be transported to other sites where it may exert its effects [17,18,19,20,21,22]. Thus, sSDC4 may have broader effects than the membrane-anchored form. It not only retains the functions of membrane-anchored SDC4, but also being freely diffusible, mediates broader paracrine actions. This soluble ectodomain can influence distal cells beyond their tissue of origin, potentially propagating OA-related pathological changes through synovial-fluid-mediated dissemination. In this review, we first review the sheddases, cleavage sites, and possible affecting factors of SDC4 shedding. Then, the functional consequences of SDC4 shedding and the potential mechanisms of sSDC4 are discussed. Finally, the possible roles of sSDC4 in OA pathogenesis are reviewed.

## 2. Proteolytic Enzymes and Cleavage Sites of SDC4 Shedding

The core protein of SDC4 comprises three distinct domains: an N-terminal ectodomain, a transmembrane domain, and a C-terminal intracellular (cytoplasmic) domain [23]. The intracellular domain of SDC4 interacts with numerous cytosolic binding partners involved in the mechanotransduction and signaling [24,25]. The ectodomain, approximately 120 amino acids in length, extends from the extracellular end to the cell membrane. This ectodomain consists of a heparan sulfate (HS)-chain-binding domain, a cell-binding domain, and proteolytic cleavage site domains [26]. SDC4 undergoes regulated proteolytic cleavage, typically near the plasma membrane, in a process known as shedding [23]. HS chains attached to the core protein inhibit the shedding of syndecan; when these HS chains are enzymatically degraded or absent, syndecan shedding is dramatically enhanced [27]. After shedding, the ectodomain of SDC4, which contains HS-chain-binding domains, can enter the ECM as an intact proteoglycan following proteolytic cleavage [28].

**Figure 1:**
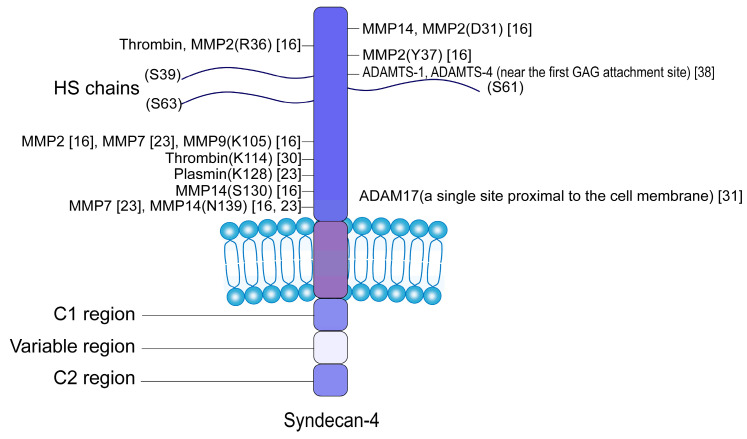
The localization of cleavage sites and heparan sulfate (HS) side chain attachment sites on the syndecan-4 (SDC4) ectodomain is illustrated. The numbers indicated represent the residue counts between cleavage sites and the galactosylated glycosaminoglycan (GAG) attachment sites. Cleavage sites are predominantly situated near the membrane. The transmembrane domain is represented as a purple bar embedded within the cell membrane. The intracellular domain comprises one variable region flanked by two conserved regions, C1 and C2.

It is currently known that SDC4 can be shed by a variety of proteolytic enzymes, including matrix metalloproteinases (MMPs) [29], plasmin [30], thrombin [30], a disintegrin and metalloproteinases (ADAMs) [31], and a disintegrin and metalloproteinase with thrombospondin motifs (ADAMTS). These enzymes are responsible for shedding the SDC4 ectodomain at specific cleavage sites in Figure 1 [32,33]. The specific shedding enzymes involved can vary among different cell types in Table 1.

## 3. Factors Affecting SDC4 Shedding

Ectodomain shedding is an important regulatory mechanism. The shedding of SDC4 occurs constitutively to a small degree as part of normal turnover. This process is accelerated under certain conditions, including inflammation, oxidative stress, mechanical stress, and the upregulated expression or activity of chemokines and cytokines, such as growth factors [39], bacterial virulence factors [40], and trypsin [15]. Different extracellular stimuli activate different intracellular signaling pathways to accelerate shedding [41].

### 3.1. Inflammation

Inflammation, mediated by tumor necrosis factor-α (TNFα) and interleukin-1β (IL-1β), can significantly upregulate the expression and shedding of SDC4. After islet beta-cells are stimulated by IL-1β, SDC4 is upregulated and shed, involving the scleroderma renal crisis-signal transducer and activator of transcription 3 (SRC-STAT3) pathway, with MMP9 potentially participating in this process [9]. Similarly, IL-1β stimulation increases the level of sSDC4 in chondrocyte culture supernatants, with MMP9 acting as the responsible sheddase [17]. Under diabetic conditions, IL-1β, rather than hyperglycemia, enhances the shedding of SDC4 from glomerular endothelial cells in an MMP9-dependent manner [36]. TNFα, IL-1β, and lipopolysaccharides (LPSs) induce SDC4 expression and shedding in cardiac myocytes and fibroblasts through the toll-like receptor 4 (TLR4) and nuclear factor kappa-B (NF-κB) signaling pathways [42]. Strand et al. discovered that IL-1β-induced upregulation of SDC4 expression in cardiomyocytes could be significantly reversed by the IL-1 receptor antagonist (IL-1Ra) in vitro [18]. Oxidized linoleic acid, the principal oxidizable fatty acid in low-density lipoprotein (LDL), and its oxidized products contribute to atherosclerosis and other chronic inflammatory cardiovascular diseases [43]. Houston et al. demonstrated that oxidized linoleic acid regulated the expression and shedding of sSDC4 on airway smooth muscle cells (ASMCs) through the generation of intracellular hydrogen peroxide and activation of the ERK signaling pathway, which might be associated with pathological processes such as atherosclerosis [43]. These findings suggest that the shedding of SDC4 is influenced by multiple inflammatory factors.

After the shedding of SDC4 in various tissues, significant amounts of soluble sSDC4 are released into body fluids, including serum. Currently, the serum levels of sSDC4 in various inflammatory diseases are summarized in Table 2. Studies have reported that serum sSDC4 levels are mainly associated with inflammation, such as pneumonia. In patients with mild pneumonia, serum sSDC4 levels were higher in comparison to those with severe pneumonia. Interestingly, short-term antibiotic therapy further elevated sSDC4 levels, leading to the hypothesis that sSDC4 might possess anti-inflammatory properties [13]. Consistently, Sato et al. observed increased serum sSDC4 levels in patients with idiopathic interstitial pneumonia [44]. Luo et al. also demonstrated that serum sSDC4 levels were associated with severe community-acquired pneumonia, and these elevated serum levels were linked to a higher mortality rate [45]. Additionally, increased serum sSDC4 levels have been reported in patients with atopic dermatitis [46] and acute myocardial infarction [47]. Serum levels of sSDC4 have thus been shown to be associated with various diseases. Therefore, differentiating the cause of elevated sSDC4 levels in different patients to determine the specific disease they relate to remains a challenge. Considering the critical role of the heparan sulfate–galactosylated glycosaminoglycan (HS-GAG) chains in cellular signaling, through their sulfated domains, HS-GAG chains mediate high-affinity binding with growth factors, cytokines, and receptors. These interactions enable direct receptor activation while sulfation patterns critically modulate fibroblast growth factor (FGF)/Wnt pathway activation. Thus, more detailed analyses of the shedding site and/or modification of HS-GAG chains may enhance the sensitivity of sSDC4 as a biomarker for the respective diseases [7]. Thus, more detailed analyses of the shedding site and/or modification of the HS-GAG chains may enhance the sensitivity of sSDC4 as a biomarker for the respective diseases [7].

### 3.2. Oxidative Stress

Oxidative stress, arising from an imbalance between reactive oxygen species (ROS) production and the antioxidant defense system [48], has been shown to affect SDC4 ectodomain shedding. It has been shown that oxidative stress can trigger the activity of ADAM-17, one of the SDC4 sheddases [49]. Silent information regulator factor 1 (*Sirtuin1*), a histone deacetylase, exerts significant antioxidative stress effects. In fibrosis-prone endothelial *Sirtuin1*-deficient (*Sirt1^endo^*^−/−^) mice, SDC4 expression was elevated via enhanced NF-κB signaling in *Sirt1*-deficient endothelial cells, and oxidative stress induced by *Sirt1* deficiency contributed to shedding of SDC4 [50]. Wu et al. found that advanced glycation end products (AGEs) could induce a senescence-associated secretory phenotype (SASP) in late-passage endothelial progenitor cells, and that resveratrol could inhibit oxidative stress and reduce ROS levels by activating *Sirtuin1*, thereby reducing SDC4 shedding induced by AGEs [51]. Xie et al. reported that AGEs promoted the SDC4 shedding through the receptor for advanced glycation endproducts (RAGE) and ROS pathways. The application of the antioxidant N-acetylcysteine (NAC) can inhibit AGE-induced SDC4 shedding, suggesting that oxidative stress plays a role in this process [52]. Moreover, increased SDC4 shedding in atrial fibrillation patients is associated with higher levels of markers of inflammation and oxidative damage, which may mediate SDC4 shedding via MMPs [53]. Increased ROS levels in OA cartilage can induce inflammation and matrix degradation in chondrocytes [48,54,55]. The relationship between oxidative stress and SDC4 ectodomain shedding in OA cartilage requires further investigation.

### 3.3. Mechanical Stress

SDC4 serves as a crucial mediator transducing mechanical stress from the ECM to chondrocytes, making mechanical stress a key factor influencing the shedding of the SDC4 ectodomain. In 2020, Jiang et al. revealed that SDC4-dependent mechanotransduction in endothelial cells is principally mediated through a dynamic scissoring mechanism of its transmembrane dimer, whereby the two extracellular domains undergo coordinated anti-parallel displacement. This scissoring motion serves as a key mechanotransduction mechanism in endothelial cells, transmitting shear stress forces from the ectodomain to the intracellular domain. Flexible linker regions enable the motion, facilitating force transfer to the cytoskeleton and downstream signaling. The heparan sulfate chains stabilize the SDC4 core protein during this process, preserving endothelial glycocalyx function [56]. Lee et al. observed exercise-induced changes in SDC4 levels among sedentary men, comparing those with dysglycemia to normoglycemic individuals. The results showed that acute exercise significantly increased serum concentrations of soluble SDC4 in both groups, suggesting that enhanced blood flow and mechanical stress during exercise may promote SDC4 shedding from the endothelial glycocalyx. This finding indicates that the observed elevation in SDC4 levels represents a physiological response to acute exercise rather than being specific to either metabolic state [20]. Abnormal mechanical loading is also a risk factor for OA [57]. The direct relationship between changes in mechanical stress changes and SDC4 shedding during the OA process remains unclear.

### 3.4. Different Specific Cytokines

Under normal physiological conditions, the shedding of HSPGs from the cell surface was not inhibited by the matrix metalloproteinase inhibitor tissue inhibitor of metalloproteinase-3 (TIMP-3). However, following abnormal stimulation, the shedding of the ectodomain was inhibited by TIMP-3 [29]. This suggests that abnormal stimuli activate different shedding enzymes and intracellular signaling pathways, consequently accelerating shedding, although the specific mechanism remains unclear.

Protein kinase C (PKC), which can be activated by phorbol esters such as phorbol myristate acetate (PMA), is a family of serine/threonine protein kinases that regulate various cellular processes including cell proliferation, differentiation, and apoptosis [58]. The shedding of SDC4 can be enhanced by PKC activation and is sensitive to peptide hydroxamate metalloproteinase inhibitors [29]. Additionally, inhibiting PKC activity prevents the PMA- and cellular-stress-induced shedding of SDC4 but does not affect the shedding activated by thrombin or the epidermal growth factor receptor. This suggests that the shedding of SDC4 mediated by the epidermal growth factor and thrombin receptors does not to involve PKC activation, which is associated with the activation of the ERK/MAPK pathway [29,39].

C-C motif chemokine ligand 5 (CCL5) is a chemokine that plays a critical role in leukocyte recruitment during inflammation. CCL5 accelerates the shedding of SDC1 and SDC4 from HeLa cells expressing C-C chemokine receptor type 5 (CCR5), and this process involves MAPK- and PKC-dependent signaling pathways [59].

Stromal cell-derived factor-1 (SDF-1), now known as C-X-C motif chemokine ligand 12 (CXCL12), is a CXC chemokine that is constitutively expressed in a wide variety of tissues. Recent research has revealed that exogenous SDF-1 demonstrates therapeutic potential in OA through activating the adenosine monophosphate-activated protein kinase (AMPK) signaling pathway in synovial cells to suppress NOD-like receptor family, pyrin domain containing 3 (NLRP3) inflammasome assembly and attenuate pyroptosis [60], while it can also restore mitochondrial homeostasis via regulation of the Sirtuin 3/peroxisome proliferator-activated receptor gamma coactivator 1-alpha (PGC-1α) signaling cascade [61], contributing to OA symptom relief. SDF1 can bind to the seven-transmembrane G protein-coupled receptor (GPCR) C-X-C chemokine receptor type 4 (CXCR4). SDF-1 promotes OA cartilage degeneration through the CXCR4 receptor, inducing chondrocyte apoptosis, necrosis, and autophagic cell death [62,63]. SDF-1 promotes osteoarthritis cartilage degeneration by activating the hypoxia-inducible factor 1 (HIF-1)/interleukin-6 (IL-6) signaling axis to induce chondrocyte ferroptosis. Specifically, SDF-1 downregulates glutathione peroxidase 4 (GPX4)/solute carrier family 7 member 11 (SLC7A11) and upregulates acyl-coA synthetase long chain family member 4 (ACSL4), leading to ROS and iron accumulation, while disrupting the MMP13/collagen type II alpha 1 (COL2A1)/aggrecan (ACAN) expression balance. These pathological changes can be reversed by the ferroptosis inhibitor Fer-1, and inhibition of the HIF-1 pathway significantly alleviates SDF-1-induced chondrocyte damage [64]. Brule et al. demonstrated that SDF-1 strongly accelerated the shedding of SDC4 from HeLa cells and human primary macrophages in a CXCR4-independent manner and that MMP-9 silencing by RNA interference significantly decreased the SDF-1-accelerated shedding of SDC4 [37].

In summary, although the shedding of SDC4 is a physiological activity, it can be accelerated by certain OA-related factors, including inflammation, oxidative stress, and mechanical stress, and regulated by various chemokines or cytokines, such as TIMP3, PMA, CCL5, and SDF1.

## 4. Functional Changes in the Remaining Membrane Domain After SDC4 Shedding

In pathological states, the activation-induced shedding of the SDC4 ectodomain results in the remaining portion of the membrane-bound receptor losing its ability to bind ligands, thereby impairing SDC4 signaling and leading to associated functional deficits [41]. For instance, in cardiac fibrosis, thrombin cleaves osteopontin (OPN), which stimulates cardiac fibroblasts to synthesize collagen. SDC4 protects against this by inhibiting OPN cleavage. However, once SDC4 is shed, this protective effect is lost, allowing fibrosis to progress [65]. SDC4 is also involved in cell migration. Upon shedding of its ectodomain, intercellular connections are disrupted, resulting in impaired cell migration. In endothelial progenitor cell (EPC) migration and homing, the ectodomain of SDC4 on EPCs can bind to SDF-1, functioning as an SDF-1 receptor and mediating EPC migration. When the ectodomain is shed, the migratory capacity of EPCs is impaired [52]. SDC4 affects the migration of endothelial cells through shedding, which contributes to the obstruction of large blood vessel formation in diabetes [10]. Conversely, the shedding of the SDC4 ectodomain can also serve as a protective mechanism. In islets, treatment with IL-1β upregulates MMP9 expression, leading to increased SDC4 proteolysis and shedding, which compromises the β-cell glycocalyx and protects them from lymphocyte infiltration [9].

## 5. Functions of sSDC4

### 5.1. sSDC4 Can Function as a Decoy Receptor, a Molecular Chaperone, and a Signaling Molecule

sSDC4 retains its original binding properties and can continue to exert biological activity as a soluble agonist or antagonist through paracrine or autocrine signaling [66]. It contains intact HS-GAG chains that retain biological activities similar to those of their parent molecule. Therefore, sSDC4 can downregulate signal transduction by competing with membrane-bound SDC4 for extracellular ligand binding and sequestering HS-binding factors in the ECM [67]. In the context of OA, SDC4 may play a critical regulatory role through its HS chains, which sequester key inflammatory mediators including TNF-α and IL-1β within the cartilage ECM. This molecular interaction maintains precise spatial control over cytokine bioavailability and contributes to the preservation of cartilage homeostasis. However, during OA progression, ECM degradation disrupts this regulatory mechanism, resulting in the pathological release of sequestered cytokines. This process establishes a vicious cycle of inflammation and cartilage catabolism, ultimately accelerating disease progression. This competitive binding allows sSDC4 to function as a decoy receptor, exerting an inhibitory effect on signaling pathways [16,41].

The sSDC4 possesses the function of a molecular chaperone. For example, under the influence of insulin, the ectodomain of SDC4 sheds from the fat cell and binds to active dimeric lipoprotein lipase (LPL) through its HS chains, thereby stabilizing LPL activity during its transcytosis from the fat cell to the endothelium [68].

The sSDC4 can act as a molecule signaling to induce changes in downstream molecules and pathways. For example, in 293T cells, ADAMTS1-mediated shedding of SDC4 may downregulate the activity of the small GTPase RhoA, a key regulator of actin fiber formation, leading to a noticeable redistribution of actin fibers to the periphery of the cell and alterations in morphology, adhesion, and migration functions [38]. In endothelial cells, knockdown of ADAMTS-1 leads to increased expression of MMP9, which in turn promotes SDC4 shedding through increased MMP9 activity and enhancing endothelial cell responsiveness to the pro-angiogenic factor vascular endothelial growth factor A (VEGFA)164, activating vascular endothelial growth factor receptor (VEGFR)2, and increasing cell proliferation and migration, thus promoting angiogenesis [69]. Additionally, either the knockdown of ADAMTS-1 or the suppression of SDC4 expression promotes the formation of fibrillar adhesions containing α5 integrin, enhancing cell migration capacity [69]. In tumorigenesis, the role of syndecan shedding has been well established [34,70,71,72,73,74,75]. For example, shedding of SDC4 can influence various biological behaviors of breast cancer cells, including cell adhesion, migration, and invasion [76], which may be associated with the progression and metastasis of breast cancer. In this process, SDC4 can influence cell migration by modulating Wnt signaling molecules. It has been demonstrated that SDC4 knockdown could reduce the expression of Wnt signaling molecules, but the impact of sSDC4 on these signaling molecules still remains to be elucidated through further experimental investigation [77].

### 5.2. sSDC4 Is Involved in the Pathogenesis of Numerous Diseases

SDC4 shedding can serve as an effector molecule in the innate immune system, participating in the innate immune regulation and chemotaxis of immune cells [19]. The excessive accumulation of SDC4 ectodomain in the ECM of *Sirt1^endo^*^−/−^ mice can act as a chemoattractant for monocytes, leading to high levels of macrophages [50]. In Th1 inflammatory conditions, sSDC4 in airway smooth muscle cells (ASMCs) may regulate chemokine activity and mast cell recruitment to the airway smooth muscle (ASM) in asthma, indirectly contributing to airway hyper-responsiveness [78]. sSDC4 can also act as a damage-associated molecular pattern (DAMP) to activate innate immune pathways [19]. It can regulate the expression of ECM molecules associated with collagen synthesis, cross-linking, and turnover in cardiac fibroblasts affecting ECM remodeling [19]. The expression and shedding of SDC4 in the heart can be induced by intraperitoneal injection of LPS in mice. SDC4 knockout mice, unable to produce sSDC4, show impaired myocardial immune cell recruitment and exacerbated cardiac dysfunction [79].

Furthermore, sSDC4 has unique roles in various diseases, but the specific mechanisms are not fully understood. Lipphardt et al. found that kidneys injected with SDC4 ectodomain exhibited greater interstitial fibrosis compared to the control kidneys in vivo, and SDC4 ectodomain stimulation of kidney fibroblasts generated myofibroblasts in vitro [50]. Thus, it is suggested that sSDC4 exerts pro-fibrotic functions in renal disease. Additionally, SDC4 shedding enhances the ability of albumin to pass through the glomerular endothelial cell monolayer [28]. In heart disease, SDC4 ectodomain fragments can increase the vascular permeability in cultured endothelial cells, which may have significant implications for inflammatory responses and repair processes following cardiac injury [80]. Additionally, shedding of the endothelial glycocalyx (EG), of which SDC4 is a structural component, is associated with poor outcomes in various conditions, including sepsis [81]. The presence of SDC4 in the supernatant is consistent with EG damage [81]. In the wound environment, sSDC4 has been detected, suggesting it may affect wound healing and tissue repair, although the specific mechanisms remain unknown [39,82].

Given its widespread expression across various tissues [83], sSDC4 has been implicated in the pathogenesis of numerous diseases (Table 3). Importantly, sSDC4 demonstrates dual functionality, acting as both a decoy receptor and a signaling molecule. These multifunctional roles highlight the complexity of its biological mechanisms. Consequently, further research is necessary to clarify the molecular interactions involving sSDC4 and to elucidate how these interactions contribute to disease progression.

## 6. The Roles of sSDC4 in OA

### 6.1. The Level of sSDC4 Is Positively Relative to the Severity of OA

The ectodomain of SDC4 plays a significant role in the progression of OA. Emerging evidence suggests that sSDC4 may serve as a potential biomarker for OA. In 2021, Bollmann et al. observed that the synovial levels of sSDC4 in patients with knee OA significantly increased with the severity of OA, while serum levels of sSDC4 remained unchanged. Using a threshold of 72.9 pg/mL, SDC4 can distinguish between early- and late-stage OA with 67.5% sensitivity and 65.2% specificity. SDC4 shedding positively correlates with MMP-9 levels, which is upregulated in OA, and MMP-9 inhibitors have been shown to reduce SDC4 shedding. Furthermore, SDC4 shedding reduces chondrocyte sensitivity to IL-1β, contributing to disease pathogenesis. These suggest that sSDC4 levels in synovial fluid could serve as a good predictor of OA severity [17]. Similarly, in an animal model of knee osteoarthritis, elevated levels of sSDC4 in the synovial fluid were also observed [85]. However, compared to established OA biomarkers—such as inflammatory markers such as IL-6 and cartilage metabolism markers like cartilage oligomeric matrix protein—the clinical advantages of SDC4 shedding require further investigation to determine its diagnostic and prognostic superiority [86]. Additionally, some studies suggest that SDC4 plays a causal role in the progression of OA [14,87,88].

### 6.2. sSDC4 Can Affect Cartilage Regeneration

The shedding of SDC4 may play a role in OA through its interaction with transforming growth factor beta (TGF-β). TGF-β binds strongly to pro-inflammatory cytokines such as IL-1β and TNFα, inhibiting their deleterious effects on cartilage [89,90]. It also stimulates the secretion of proteoglycans and type II collagen from chondrocytes, which are essential for cartilage regeneration and repair [91,92]. Activation of the TGF-β/Smad signaling pathway can reduce joint inflammation, promote chondroregeneration, and slow the progression of OA. However, sSDC4, which binds to TGF-β and inhibits the activation of Smad2 and Smad3, suppresses the TGF-β/Smad signaling pathway, potentially promoting inflammation and impeding the regeneration and repair of articular cartilage [93]. Injection of anti-SDC4 antibodies may block the interactions of SDC4 on chondrocytes and sSDC4 in synovial fluid with cytokines such as ADAM metallopeptidase with thrombospondin type 1 motif 5 (ADAMTS-5) and IL-1, thereby inhibiting cartilage degradation and promoting cartilage regeneration [14]. Similarly, another study demonstrated that anti-SDC4 antibody injection upregulated miR-96-5p in chondrocytes, which targeted HIF-2α 3’-UTR sequences and suppressed HIF-2α signaling in murine cartilage tissue and chondrocytes [88]. However, a study in 2023 reported that intra-articular SDC4 injection decreased ADAMTS-5 expression and increased TIMP-3 expression, suggesting that SDC4 treatment inhibits cartilage degeneration in osteoarthritic articular cartilage, potentially enhancing cartilage regeneration [87]. These conflicting findings may be attributed to differences in experimental design, such as the concentration of sSDC4 used in the injections. Anyway, before intra-articular injection of anti-SDC4 antibodies, the precise role of sSDC4 in OA pathogenesis remains to be fully elucidated and warrants further investigation.

### 6.3. sSDC4 Can Act as a DAMP to Destroy the Cartilage Homeostasis

Necroptosis was the primary form of cell death in the early stage of condylar cartilage degeneration in a rat model of temporomandibular joint osteoarthritis (TMJOA). During necroptosis, SDC4 shedding occurred, and sSDC4 was released into synovial fluid in vivo or the cell supernatant in vitro. Furthermore, the stimulation of chondrocytes with the SDC4 ectodomain led to significantly higher levels of TNFα, ADAMTS5, and MMP13 in both chondrocytes and supernatants in vitro. In addition, blocking SDC4 with a specific antibody downregulated the expression of TNFα, ADAMTS5, and MMP13. Thus, sSDC4 released after chondrocyte necroptosis might independently induce subsequent cartilage degeneration. As expected, blocking SDC4 function significantly attenuated TNFα expression in cartilage and synovium, thereby recovering cartilage thickness and reducing synovial inflammation. Collectively, SDC4 might act as a key DAMP that forms a necroptotic vicious cycle of TNFα-SDC4-TNFα, leading to condylar cartilage degeneration and synovitis [94].

## 7. Conclusions and Perspectives

**Figure 2:**
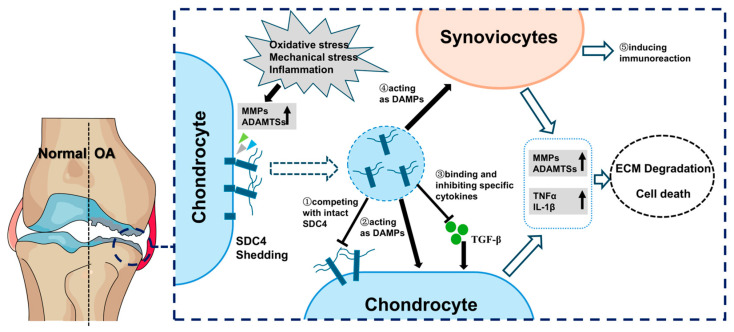
The roles of shed syndecan-4 (sSDC4) in osteoarthritis (OA).

In summary, under normal physiological conditions or in response to pathological stimuli such as inflammation, oxidative stress, or mechanical stress, various proteases in cartilage (including MMPs and ADAMTSs) cleave SDC4 at specific sites, leading to the shedding of its ectodomain into the ECM. Upon shedding of the SDC4 ectodomain, the remaining membrane-bound receptor loses its ability to bind ligands, thereby impairing extracellular and intracellular SDC4 signaling and disrupting the normal interaction between chondrocyte and the ECM. On the other hand, soluble sSDC4, which retains its binding properties, can function as a paracrine or autocrine signaling molecule. In local cartilage, sSDC4 can downregulate signal transduction by competing with membrane-bound SDC4 for extracellular ligand binding and sequestering HS-binding factors in the ECM. Additionally, sSDC4 can function as a decoy receptor by competing with intact SDC4 to exert an inhibitory effect. Furthermore, soluble sSDC4 released into synovial fluid or serum can act as a DAMP to stimulate chondrocytes, synovial fibroblasts, macrophages, or other immune cells, leading to degenerative, inflammatory, or immunoreactive changes. The level of sSDC4 in synovial fluid is positively correlated with the severity of OA. Thus, the shedding of SDC4 may play a critical role in the pathogenesis and progression of OA, as shown in Figure 2, although the underlying mechanism remains unclear. Although pain is a frequent manifestation of OA, its mechanisms remain poorly understood, and the potential involvement of SDC4 in pain has received minimal research attention [95]. SDC4 interacts with fibronectin (FN), a crucial extracellular matrix protein, to regulate key signaling molecules including protein kinase C epsilon (PKCε), focal adhesion kinase (FAK), and ERK1/2. This SDC4-FN interaction mediates cell adhesion, mechanotransduction, and cytoskeletal remodeling, ultimately modulating the growth, morphology, and mechanical sensitivity of dorsal root ganglion (DRG) neurons [96]. Given that DRG neurons are essential for transmitting pain signals to the central nervous system, these findings suggest that SDC4 may contribute to OA pain pathogenesis by altering DRG neuron structure and function. However, further investigation on this mechanism, or the effect of sSDC4 on nervous tissues or the expression of pain related cytokines in joint tissues, is warranted to fully elucidate it [97]. To mitigate the effects of the shedding of SDC4 or sSDC4, therapeutic strategies may include the use of specific protease inhibitors to block SDC4 shedding or the intra-articular injection of anti-sSDC4 antibodies. Thus, in future studies, elucidating the specific molecular mechanisms of SDC4 shedding in the OA process and the detailed and main effect of sSDC4 on the OA process will help to identify and validate potential therapeutic targets, providing new strategies for effective OA treatment.

## Figures and Tables

**Table 1:** Different sheddases’ cleavage sites on SDC4 and the identified cell types.

Sheddases	Cleavage Sites	Cell Types	Diseases
MMP2	Asp(31)-Leu(32) [16] Arg(36)-Tyr(37) [16] Tyr(37)-Phe(38) [16] Lys(105)-Leu(106) [16]	Mouse brain microvascular endothelial cells	Gliomas [34]
MMP7	Lys(105)-Leu(106) [23] Asn(139)-Ile(140) [23]	B-lymphocytes	Inflammation caused by infection [35]
MMP9	Lys(105)-Leu(106) [16]	Chondrocytes, glomerular endothelial cells, pancreatic β-cells, HeLa cells and human primary macrophages, mouse brain microvascular endothelial cells	Osteoarthritis [17] Diabetic nephropathy [28,36] Type 1 diabetes [9] Tumor [37] Gliomas [34]
MMP14 (MT1-MMP)	Asp(31)-Leu(32) [16] Ser(130)-Met(131) [16] Asn(139)-Ile(140) [16,23]	--	--
Plasmin	Lys(128)-Val(129) [23]	Human umbilical vein endothelial cells	Atheriosclerosis [30]
Thrombin	Arg(36)-Tyr(37) [16] Lys(114)-Arg(115) [30]	Human umbilical vein endothelial cells	Atheriosclerosis [30]
ADAMTS-1 and -4	N-terminus near the first GAG attachment site	Mouse lung endothelial cells	Atheriosclerosis [38]
ADAM17	A single site proximal to the cell membrane	the bladder carcinoma epithelial cell line ECV304, the lung epithelial cell line A459, primary alveolar epithelial cells	Acute lung injury [31]

MMP2, matrix metalloproteinase 2; MMP7, matrix metalloproteinase 7; MMP9, matrix metalloproteinase 9; MMP14, matrix metalloproteinase14; MT1-MMP, membrane-type 1 matrix metalloproteinase; ADAMTS-1, a disintegrin and metalloproteinase with thrombospondin-type motifs-1; ADAMTS-4, a disintegrin and metalloproteinase with thrombospondin-type motifs-4; GAG, galactosylated glycosaminoglycan; ADAM17, a disintegrin and metalloprotease 17.

**Table 2:** Serum levels of sSDC4 in inflammatory diseases.

Disease	Health Controls	Numbers	Age (Years)	Sex (M/F)	Patients	Numbers	Age (Years)	Sex (M/F)	Reference
Acute pneumonia	15.10 ± 2.60 ng/mL	11	50.1 ± 4.8	6/5	20.3 ± 8.9 ng/mL	30	67.1 ± 3.1	20/10	[13]
Idiopathic interstitial pneumonia	16.05 ± 0.77 ng/mL	45	43 ± 2	29/16	Clinically stable idiopathic interstitial pneumonia: 25.22 ± 3.72 ng/mL	62	68 ± 1	47/15	[44]
					Acute exacerbation of idiopathic interstitial pneumonia: 10.65 ± 0.73 ng/mL	56	70 ± 1	47/9	
Community-acquired pneumonia	14.30 ± 5.34 ng/mL	30	--	--	Non-severe community-acquired pneumonia: 10.15 ± 4.37 ng/mL	149	50 (33–65)	92/57	[45]
					Severe community-acquired pneumonia: 9.54 ± 5.92 ng/mL	103	56.14 ± 17.32	73/30	
Atopic dermatitis	0.71 ± 0.67 ng/mL	56	33.8 ± 13.13	29/27	1.41 ± 1.06 ng/mL	59	32.1 ± 12.56	32/27	[46]
Osteoarthritis	0.019 ± 1.36 ng/mL	16	--	--	19.23 ± 0.92 ng/mL	29	--	--	[17]

**Table 3:** Role of sSDC4 in disease pathogenesis.

Disease/Condition	Involvement of sSDC4	Reason for Involvement	Organ/System Affected	Reference
Unilateral Ureteral Obstruction (UUO)	Increased sSDC4 levels in the extracellular matrix, contributing to renal fibrosis.	1. Enhanced NF-κB signaling leading to increased SDC4 expression. 2. Oxidative-stress-induced shedding of sSDC4. 3. sSDC4 acts as a chemoattractant for monocytes/macrophages. 4. The ectodomain of SDC4 promotes the transition of renal fibroblasts to myofibroblasts and enhances the synthesis of the extracellular matrix. 5. Injection of the SDC4 ectodomain leads to an increase in renal fibrosis.	Kidney	[50]
Tubulointerstitial Fibrosis	SDC4 knockout reduces fibrosis, indicating a pro-fibrotic role of sSDC4.	1. SDC4 ectodomain promotes fibroblast-to-myofibroblast transition. 2. SDC4 ectodomain enhances collagen cross-linking.	Kidney	[50]
Diabetic Nephropathy	TNF-α-induced SDC4 shedding leads to increased glomerular permeability and proteinuria.	1. TNF-α activates MMP9, leading to SDC4 shedding. 2. Loss of sSDC4 disrupts the glomerular endothelial glycocalyx, increasing protein permeability.	Kidney	[28]
Endothelial Dysfunction	Elevated sSDC4 in the secretome of endothelial cells, contributing to fibrogenesis.	1. *Sirt1* deficiency leading to increased NF-κB activity and SDC4 expression. 2. Oxidative stress causing increased shedding of SDC4.	Vascular system	[50]
Myocardial Infarction	sSDC4 plays a role in wound healing and fibrosis following myocardial infarction.	sSDC4 promotes collagen cross-linking and immune cell infiltration.	Heart	[84]
Asthma	Increased expression and shedding of SDC4 in airway smooth muscle cells (ASMCs).	1. IL-1β and TNF-α enhance SDC4 shedding, which may regulate chemokine activity and mast cell recruitment. 2. sSDC4 binds chemokines like CXCL 10, potentially modulating their activity and contributing to airway hyper-responsiveness and remodeling. 3. Asthmatic ASMC may concentrate more SDC4 on their surface, leading to increased local chemokine accumulation.	Lungs (specifically airway smooth muscle)	[78]
Septic Cardiac Dysfunction	Increased expression and shedding of SDC4 in cardiac myocytes and fibroblasts.	1. LPS challenge induces NF-κB-dependent upregulation of SDC4 expression and shedding. 2. Shed SDC4 ectodomains promote the expression of adhesion molecules and cytokines, facilitating immune cell recruitment to the myocardium. 3. Shed SDC4 regulates the expression of ECM components, including collagen synthesis and cross-linking enzymes, affecting cardiac stiffness and function.	Heart	[19]
Heart Failure	Elevated circulating levels of sSDC4 in patients.	1. Chronic inflammation in heart failure leads to increased SDC4 shedding, which may reflect ongoing cardiac remodeling. 2. sSDC4 levels correlate with cardiac remodeling parameters and could serve as a biomarker for disease severity.	Heart	[19]
Acute Myocardial Infarction	Increased plasma levels of sSDC4; sSDC4 deficiency exacerbates cardiac dysfunction and impairs immune cell recruitment.	1. SDC4 shedding is part of the acute inflammatory response following myocardial infarction. 2. sSDC4 may play a role in the recruitment of immune cells and ECM remodeling during the healing process.	Heart	[19,79]
Thrombotic Diseases	Increased shedding of SDC4.	Elevated thrombin activity during thrombotic events leads to SDC4 cleavage, contributing to endothelial barrier dysfunction.	Cardiovascular system	[80]
Sepsis	Increased shedding of SDC4.	Elevated levels of inflammatory mediators lead to endothelial glycocalyx damage and SDC4 shedding.	Multiple organs (systemic involvement)	[81]
Wound	Increased shedding of SDC4.	Unknown.	Skin	[82]

## Abbreviations

The following abbreviations are used in this manuscript:

ADAMs	a disintegrin and metalloproteinases
ADAMTS	a disintegrin and metalloproteinase with thrombospondin motifs
AGEs	advanced glycation end products
ASM	airway smooth muscle
ASMC	airway smooth muscle cells
AMPK	adenosine monophosphate-activated protein kinase
ACSL4	acyl-coA synthetase long chain family member 4
ACAN	aggrecan
CCL5	C-C motif chemokine ligand 5
CCR5	C-C chemokine receptor type 5
CXCR4	C-X-C chemokine receptor type 4
CXCL12	C-X-C motif chemokine ligand 12
COL2A1	collagen type II alpha 1
DAMP	damage-associated molecular pattern
DRG	dorsal root ganglion
ECM	extracellular matrix
EPC	endothelial progenitor cell
EG	endothelial glycocalyx
ERK	extracellular regulated protein kinases
FN	fibronectin
FAK	focal adhesion kinase
FGF	fibroblast growth factor
VEGFA	vascular endothelial growth factor A
VEGFR	vascular endothelial growth factor receptor
GAG	galactosylated glycosaminoglycan
GPCR	G protein-coupled receptor
GPX4	glutathione peroxidase 4
HSPGs	heparan sulfate proteoglycans
HS	heparan sulfate
HIF-2α	hypoxia-inducible factor 2 alpha
HIF-1	hypoxia-inducible factor 1
IL-1β	interleukin-1β
IL-1Ra	IL-1 receptor antagonist
IL-6	interleukin-6
LPS	lipopolysaccharides
LDL	low-density lipoprotein
LPL	lipoprotein lipase
LRP	low-density lipoprotein receptor-related protein
MMPs	matrix metalloproteinases
MAPK	mitogen-activated protein kinase
NAC	N-acetylcysteine
NF-κB	nuclear factor kappa-B
NLRP3	NOD-like receptor family, pyrin domain containing 3
OA	osteoarthritis
OPN	osteopontin
PKC	protein kinase C
PMA	phorbol myristate acetate
PKCε	protein kinase C epsilon
PGC-1α	peroxisome proliferator-activated receptor gamma coactivator 1-alpha
ROS	reactive oxygen species
RAGE	receptor for advanced glycation endproducts
SDC4	syndecan-4
SDC	syndecan
sSDC4	shed syndecan-4
SRC	scleroderma renal crisis
STAT3	signal transducer and activator of transcription 3
*Sirtuin1*	silent information regulator factor 1
*Sirt1^endo^*^−/−^	endothelial sirtuin1-deficient
SASP	senescence-associated secretory phenotype
SDF-1	stromal cell-derived factor-1
SLC7A11	solute carrier family 7 member 11
TNFα	tumor necrosis factor-α
TIMP	tissue inhibitor of metalloproteinase
TLR4	toll-like receptor 4
TGF-β	transforming growth factor beta
TMJOA	temporomandibular joint osteoarthritis
UUO	Unilateral Ureteral Obstruction
